# Volcanic activity controls cholera outbreaks in the East African Rift

**DOI:** 10.1371/journal.pntd.0008406

**Published:** 2020-08-10

**Authors:** Doudou Batumbo Boloweti, Patrick Giraudoux, Catherine Deniel, Emmanuel Garnier, Frederic Mauny, Celestin Mahinda Kasereka, Roger Kizungu, Jean Jacques Muyembe, Didier Bompangue, Gudrun Bornette

**Affiliations:** 1 UMR CNRS 6249 Chrono-Environnement, University of Bourgogne Franche Comté, Besançon, France; 2 UMR CNRS 6249 Chrono-Environnement, University of Bourgogne Franche Comté, Besançon, France; 3 UMR CNRS 6524 Laboratoire Magmas et Volcans, University of Blaise Pascal-CNRS-IRD, Clermont Ferrand, France; 4 UMR CNRS 6249 Chrono-Environnement, University of Bourgogne Franche Comté, Besançon, France; 5 UMR CNRS 6249 Chrono-Environnement, University of Bourgogne Franche Comté, Besançon, France; 6 Volcano Observatory of Goma (VOG), Goma, Democratic Republic of Congo; 7 Faculty of Agronomy, University of Kinshasa, Kinshasa, Democratic Republic of Congo; 8 Department of Microbiology, Faculty of Medicine, University of Kinshasa, Kinshasa, Democratic Republic of Congo; 9 UMR CNRS 6249 Chrono-Environnement, University of Bourgogne Franche Comté, Besançon, France; 10 UMR CNRS 6249 Chrono-Environnement, University of Bourgogne Franche Comté, Besançon, France; Yale University Yale School of Public Health, UNITED STATES

## Abstract

We hypothesized that Cholera (*Vibrio cholerae*) that appeared along Lake Kivu in the African Rift in the seventies, might be controlled by volcano-tectonic activity, which, by increasing surface water and groundwater salinity and temperature, may partly rule the water characteristics of Lake Kivu and promote *V*. *cholerae* proliferation. Volcanic activity (assessed weekly by the SO_2_ flux of Nyiragongo volcano plume over the 2007–2012 period) is highly positively correlated with the water conductivity, salinity and temperature of the Kivu lake. Over the 2007–2012 period, these three parameters were highly positively correlated with the temporal dynamics of cholera cases in the Katana health zone that border the lake. Meteorological variables (air temperature and rainfall), and the other water characteristics (namely pH and dissolved oxygen concentration in lake water) were unrelated to cholera dynamics over the same period. Over the 2016–2018 period, we sampled weekly lake water salinity and conductivity, and twice a month vibrio occurrence in lake water and fish. The abundance of *V*. *cholerae* in the lake was positively correlated with lake salinity, temperature, and the number of cholera cases in the population of the Katana health zone. *V*. *cholerae* abundance in fishes was positively correlated with *V*. *cholerae* abundance in lake water, suggesting that their consumption directly contaminate humans. The activity of the volcano, by controlling the physico-chemical characteristics of Lake Kivu, is therefore a major determinant of the presence of the bacillus in the lake. SO2 fluxes in the volcano plume can be used as a tool to predict epidemic risks.

## Author summary

The area of the African Great Lakes has been an endemic area for cholera since the late 1970s. We focused on the Katana health zone, bordering Lake Kivu, as during outbreaks, this is, (together with the Kalemie health zone located along the west coast of the Tanganyika lake) the health zone in which the first cases of Cholera are usually observed, and the highest number of cases are also usually reached in this area. The persistence of this aquatic bacillus, usually associated with warm and salty waters, led us to formulate the hypothesis that the geothermal springs supplying Lake Kivu, mainly from the Nyiragongo volcano, should control the physico-chemical characteristics of the lake and promote the persistence of the bacillus. The lake would thus be a reservoir of the pathogen, which could contaminate local residents through the consumption of water and fish. Over the 2007–2012 period, we demonstrated a long-term unidirectional relationship between volcanic activity and cholera cases in the Katana health Zone. Contamination of the lake's water and fish was also correlated to the lake characteristics. The activity of the volcano can thus be used for predicting epidemic risks.

## Introduction

Cholera is a severe infectious disease caused by *Vibrio cholerae*. This bacillus thrives in alkaline saline aquatic environments with high temperatures and rich in organic matter and plankton [1–6]. The bacillus is found in coastal areas, where fresh water from rivers mixes with salt water from the sea [1, 2, 7–10]. Contamination frequently occurs via the consumption of water, fish, and other foods containing cholera bacilli [11–13]. The disease was introduced to continental Africa in 1970, during the seventh pandemic. It became endemic far from coastal areas, particularly in the Lake Chad basin and the Great Lakes region [14–16]. The Great Lakes are highly suspected of being reservoirs for the cholera bacillus, while human infection and movement are considered to propagate the disease inland [17]. However, studies have failed to identify the physico-chemical parameters of the lakes or/and socio-anthropological conditions that may explain cholera persistence [18, 19]. A significant role of meteorology has been found, with rainfall increasing the number of cholera cases, e.g., in Kivu province, but without excluding the role of population movements [20].

**Fig 1:**
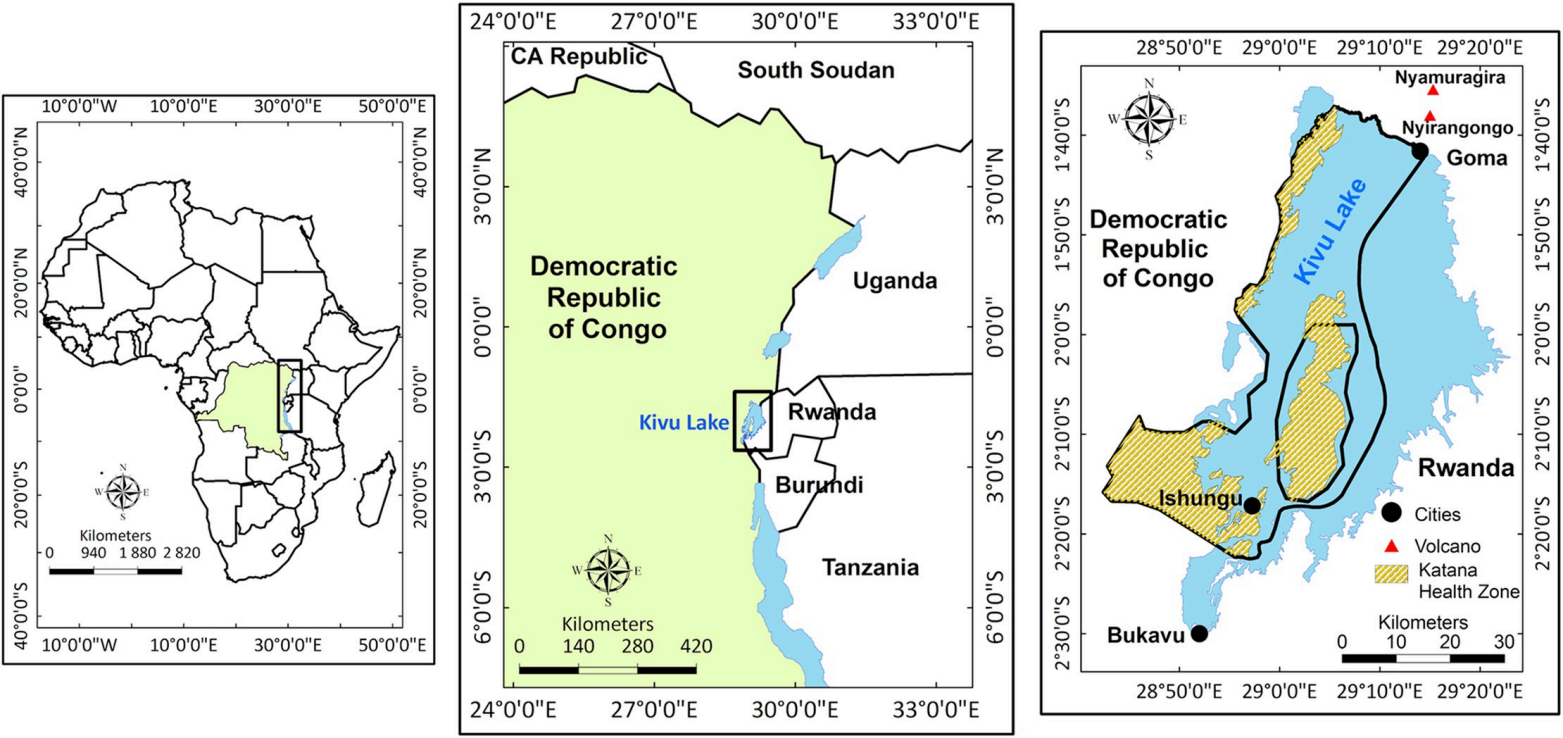
Map of the Katana health zone bordered by Kivu Lake, the two large active volcanoes in the area are shown (right) and other Lakes in the African Rift Valley (middle). This map was created using QGIS version 2.18 (http://qgis.org).

Tectonic and volcanic activities are strongly coupled in the Kivu area of the Rift (Fig 1). Indeed, Lake Kivu is located in the western branch of the East African Rift, south of the Nyiragongo and Nyamuragira volcanoes. Volcanic plumes from these very active, strongly alkaline volcanoes produce very large amounts of acidic gases and ash, which have significant direct and indirect impacts on lake surface water via interactions between acidic rain and rocks that modify the physico-chemical characteristics of rivers and groundwaters before they reach Lake Kivu [21–26]. In addition, sub-groundwaters clearly originating from hydrothermal sources linked to the major NE-SW fault system between the Nyiragongo crater and Lake Kivu also discharge into the lake [22, 27–29]. Especially in the north-western region and Kabuno Bay, Lake Kivu is supplied by hydrothermal sub-groundwaters from a deep contiguous geothermal reservoir associated with major faults [29, 30].

Volcano-tectonic activity, by controlling the salinity and/or temperature of both streams and groundwaters that feed the lake, might control the salinity and temperature of the lake, the occurrence of *Vibrio* in water and fish, and cholera dynamics. To test this hypothesis, we used 2 data sets. Firstly, using 6 years of data collected weekly from 2007 to 2012, we modelled the statistical relationships between the long-term dynamics of cholera cases in the Katana health zone and 1) the intensity of the volcanic activity, assessed through the SO_2_ flux measured in the Nyiragongo plume; 2) rainfall and air temperature; and 3) the physico-chemical characteristics of the surface water of Lake Kivu, including temperature and salinity, which are usually considered to be the main parameters controlling *Vibrio* growth. Second, using 2 years of data collected in 2016 and 2017, we modelled the relationships between the temperature and salinity of the lake water, *Vibrio* occurrence and abundance in the lake (water and fish), and the number of cholera cases in the Katana health zone, which borders the lake close to the Nyiragongo volcano.

## Materials and methods

### Study area and data

The Katana Health Zone is located in Kabare territory, in South Kivu province, in the eastern part of the Democratic Republic of Congo (DRC) (Fig 1). It covers the territory located along the west and north coast of Lake Kivu, together with the islands of the lake. The Katana health zone was selected because it was one of the first zones affected by cholera in 1978 in the rift valley. Furthermore, the Katana health zone is (together with the Kalemie health zone located along the west coast of the Tanganyika lake), the health zone in which the first cases of Cholera are usually observed, and the highest number of cases are also usually reached in this area [31]. Lake Kivu is supplied by many hydrothermal groundwater springs and highly mineralized rivers [30, 32, 33]. The northern basin of the lake is located at the end of a deep, major NE-SW fracture zone originating at the Nyiragongo crater and closely associated with volcanic (including hydro volcanism) and hydrothermal activity.

Two data sets were used for testing the hypothesis. A first data set grouped data collected twice a month over the 2007–2012 period: the number of cholera cases in the Katana health zone, meteorological data, physico-chemical characteristics of the lake water, and the SO_2_ flux (Tonnes/day) of the Nyiragongo plume as a proxy of the intensity of volcanic activity (S1 Table).

We documented a second data set that grouped data collected over the 2016–2017 period: water temperature and salinity measured weekly in the surface water of the lake, the number of cholera cases in the Katana health zone, and the SO_2_ flux (Tonnes/day) of the Nyiragongo plume as a proxy of the intensity of volcanic activity, and the abundance of *Vibrio* in the lake’s surface water and fishes measured twice per month (S2 and S3 Tables).

### Geochemistry of volcanic gases

The SO_2_ flux, which directly reflects the intensity of magmatic degassing in the Nyiragongo crater, was chosen as a proxy of the intensity of volcanic activity. Quantification of the SO_2_ flux of the Nyiragongo plume was performed continuously by remote sensing UV absorption spectroscopy (280–420 nm) with telemetric data transmission [25, 34–36]. Data covering the study period were obtained at the Rusayo station, located on the southwestern flank of the Nyiragongo volcano and belonging to the database of the Volcanological Observatory of Goma (VOG). Mean of SO_2_ values were calculated for each week to be compared with the other data.

### Physico-chemical characteristics of Lake Kivu

Water temperature, pH, electrical conductivity, and dissolved oxygen potentially regulate *V*. *cholerae* [20, 37, 38]. Over the 2007–2012 period, these parameters were measured twice per month in surface water collected in the lake near Ishungu. Water temperature and electrical conductivity were measured using a portable conductivity meter (HACH CO150, Hach Company, USA). Dissolved oxygen and pH were measured using a HACH portable dissolved oxygen meter (HACH DO175, Hach Company, USA) and an Orion pH meter (Model 210A, Orion Laboratories), respectively. Air temperature and rainfall, which are frequently considered to be key factors affecting cholera outbreaks, were compiled over the same time period. These observational data were obtained from the Modern-Era Retrospective Analysis for Research and Applications (MERRA) of the National Aeronautics and Space Administration (NASA).

Over the 2016–2017 period, temperature was measured weekly in the same place using a portable conductivity meter. Salinity was measured using water collected at the same place using a salinity kit (Chloride CL 500, Visocolor HE).

### *V. cholerae* occurrence in water and fishes

Twice a month over the 2016–2017 period, the abundance of *V*. *cholerae* in the lake was assessed in surface lake water samples collected near Ishungu (same location and sampling dates as salinity and temperature). We filtered one litre of water using a 0.22 μm-pore size sterile polycarbonate membrane filter. If the sample was too turbid, several membranes were used. The filters were then put in Alkaline Peptone Water (APW; Difco, Detroit, MI, USA) and incubated at 37°C for 24 h. Five microlitres of enriched APW broth were then streaked with an inoculating loop onto plates containing Thiosulfate Citrate Biliary Salts Sucrose (TCBS) ([39], American Public Health Association, American Water Works Association, Water Pollution Control Federation 1999). The plates were incubated at 37°C for 16 to 24 h in TCBS. Yellow, flat, 1–3 mm diameter colonies were counted and expressed as the number of colonies per 100 ml (number of Colony Forming Units (CFUs) per 100 ml).

For *V*. *cholerae* identification, suspected colonies were transplanted onto non-selective Alkaline Nutrient Gelose (ANG) agar and incubated at 37°C for 24 h. The Gelatin-positive cultures were subjected to an oxidase test. Oxidase-positive colonies were then subjected to further biochemical characterization using an API 20E gallery.

The search for *V*. *cholerae* in fishes was carried on the same dates as water sampling over the 2016–2017 period. Six fishes were collected twice a month from the Ishungu Basin. A total of 288 fish were sampled and brought to the laboratory, where intestinal and gill samples were systematically collected and checked for *V*. *cholerae* occurrence. One gram of fish sample was inoculated into a tube filled with 9 ml of APW. The tube was vortexed, and its content was then successively diluted in three tubes to 1/10, 1/100 and 1/1000. The exact volume used for dilution allowed us to express the result as the number of colonies per gram of sample. Then, 0.1 ml of each dilution was plated onto TCBS agar and incubated overnight at 37°C. Yellow colonies on the TCBS medium suspected of being *V*. *cholerae* were counted and transplanted onto lysogeny broth agar for identity confirmation. The final number of colonies was counted and expressed in CFUs per gram of intestine or gill (see microbiological protocol for more details).

### Epidemiological Data

Weekly notifications of cholera cases in the Katana zone were used as epidemiological data. The cases of cholera were defined according to the recommendations of the World Health Organization (WHO). These epidemiological data covered the periods of 2007–2012 and 2016–2017.

### Statistical analyses

We first searched for a significant correlation between the number of cholera cases (2007–2012 period) and 1) the SO2 flux, 2) lake water characteristics and 3) meteorological data. We analysed the data by week, incorporating 147 time-points during the study period (2007–2012). Direct and lagged cross-correlations between each environmental variable and the log-transformed number of cholera cases (transformation done to normalize the data) were computed and tested using Pearson's correlation coefficient. We verified that log-transformation led to a normal distribution of the variable “number of cholera cases”. Moreover, we visually verified that the assumption of linearity was not abusive for the different relationships tested. Considering that data might be auto correlated to some degree (they are parts of a time series), p(H_0_) was computed using permutation tests. Then, highly correlated and statistically significant predictors were selected to construct a multivariate time series Vector Auto Regressive model (VAR) [40, 41], which allow to test the correlations between several time series. A VAR model describes the evolution of a set of k variables (called endogenous variables) over the same sampling period as a linear function of their past values. It is a natural extension of the univariate autoregressive model to dynamic multivariate time series. It also determines how each endogenous variable responds over time to a shock (aberrant behaviour due to sudden and unexpected events) in its own value and in every other variable [41, 42].

The basic form of the VAR model of order p as suggested by [43] has the form:

$$Y_{t}=A_{0+}A_{1}Y_{t-2}+A_{2}Y_{t}{}_{-2}+\cdots +A_{p}y_{t}{{}_{-}}_{p}+\varepsilon_{t}$$

Where:

Y_𝑡_ = (𝑦_1𝑡_, 𝑦_2𝑡_,…, 𝑦_𝑘𝑡_)’ is a vector of k observable endogenous variables and p the time lag. For this study, 𝑦_𝑡_ = (Cholera_𝑡_, 𝑇_t_, Rain_𝑡_, Cd_t_, pH_t_, SO_2t_)’, where Cholera represents the number of cholera cases every two weeks, 𝑇 represent the lake temperature (°C), Rain is the rainfall (mm), Cd is the water conductivity (μS/cm), pH is water pH, and SO_2_ is sulphur dioxide concentration of the volcanic plume (Tonnes/day). A_0_ is vector of constant term and ε_t_ is vector of error terms.

The parameters in the model were estimated by generalised least squares. We tested the stationarity of each time series (cholera cases, water temperature, rainfall, conductivity, pH and the SO2 volcanic flux) to be included in the model with the Augmented Dickey Fuller Test (ADF). The number of lags was chosen based on four tests: The Final Prediction Error (FPE) test [44], the Hannan Quinne (HQ) test [45], and the Information Criteria suggested by Akaike (AIC) [46] and by Schwarz (SC) [47]. The parameters in the model were estimated by generalised least squares. Despite the fact that VAR coefficients capture the anticipated impact of a variable, there are usually more important to examine the model residuals, which represent unforeseen contemporaneous events. Thus, to examine the fit of the model, we performed diagnostic tests on the residuals of the model. To test the correlation of series, we applied the Portmanteau test. To test for heteroscedasticity in the residuals, we used a multivariate ARCH Lagrange-Multiplier test. To consider the distribution of residues, a normality test was applied. Both the Granger-causality and instantaneous causality were investigated. For both tests, the vector of endogenous variables was divided into two subvectors, Y_1𝑡_ 𝑎𝑛𝑑 Y_2𝑡_, with dimensions k_1_ 𝑎𝑛𝑑 k_2_, respectively, so that k = k_1_+k_2_. The subvector 𝑦_1𝑡_ was said to be Granger-causal for 𝑦_2𝑡_ if the past of 𝑦_1𝑡_ significantly helped predicting the future of 𝑦_2𝑡_ via the past of 𝑦_1𝑡_ alone [48]. We then tested the impulsive responses to describe the response of the cholera incidence to the various predictor shocks.

Second, for the data set covering the 2016–2017 period and incorporating 48 time-points, we looked for significant correlations between lake temperature and salinity and 1) the SO2 flux, 2) the V. *cholerae* concentration in water and fish, 3) cholera cases, and 4) the *V*. *cholerae* concentration in water and fish, and cholera cases using direct and lagged cross-correlations. The small size of this second data package did not allow the multiple regression models of time series to be developed.

Difference of contamination between intestines and gills were analysed using a linear mixed effect model with intestine contamination as the response variable and gill contamination as independent variable, with a random effect on time. Sampling over time might lead to some degree of autocorrelation along time series, and subsequent non-independence of data (with possible overestimation of degrees of freedom). This was avoided using a permutation test based on 1000 replicates for testing the null hypothesis of independence between intestine and gill contamination.

All tests were two-sided, and the level of statistical significance was set at 0.05. Computing and graphing were performed using R 3.4.4.

The study used cholera data aggregated at the health-zone level; thus, it did not require any ethical review board approval.

## Results

Summary of the data set and descriptive statistics are presented in Table 1.

**Fig 2:**
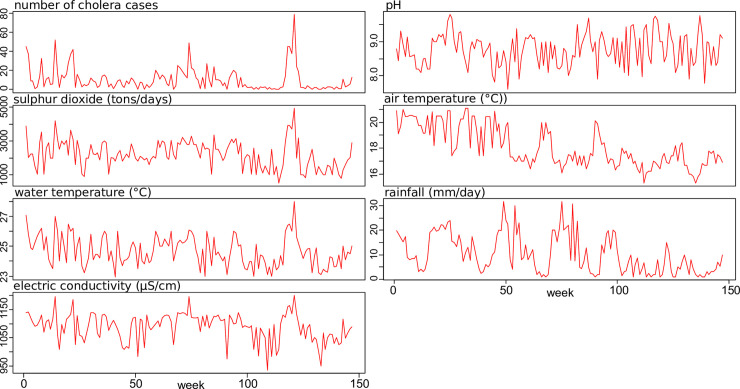
Time series plot of cholera cases and environmental variables for the period of 2007 to 2012, Katana health zone, DR Congo (n = 147).

Time series plots of cholera cases and of environmental variables collected from 2007 to 2012 are presented in Fig 2. The global correlation analysis of the data set covering the period of 2007–2012 showed that the sulphur dioxide concentration of the volcanic plume was strongly and positively correlated with lake water temperature and conductivity, and cholera cases. Water temperature and conductivity were also strongly and positively correlated with the number of cholera cases. The other correlations were much weaker, but some were significant (Table 2).

**Table 1:** Summary of data collected during the two sampling periods.

Period	Variables	Median	Minimum	Maximum
2007–2012 (147 sampled dates)	Number of cholera cases (cases/week)	6	0	79
	Rainfall (mm/week)	8	1	31.6
	Average air temperature (°C)	17.5	15.3	21.1
	SO_2_ (tons/day)	2108	512	4952
	Electric conductivity (μS.cm^-1^)	1098	935	1200
	Water temperature (°C)	24.6	22.9	28
	pH	8.8	7.6	9.8
	Dissolved O_2_ ([O_2_] mg.L^-1^)	7.1	4.5	10.6
2016–2017 (48 sampled dates)	Water temperature (°C)	24.2	22	27
	Salinity (‰)	0.87	0.65	1.16
	*V*. *cholerae* in water (cfu/100 ml)	3000	0	23000
	*V*. *cholerae* in fish (cfu/g)	505	0	16600
	SO_2_ (tons/day)	1350	800	4000
	Number of cholera cases (cases/week)	4	0	48

**Table 2:** Pearson correlation coefficients between logarithmic transformation of observed number of cholera cases and environmental variables over the 2007–2012 period (n = 147 sampled dates).

Variables	Cholera cases (/week)	Rainfall (mm/week)	Average air temperature (°C)	SO_2_ (tons/day)	Electric conductivity (μS.cm-1)	Water temperature (°C)	pH	Dissolved O_2_ (mg.L-1)
Cholera cases (/week)		0.32*	0.26*	0.73**	0.80**	0.82**	0.28*	-0.04
Rainfall (mm/week)			0.21*	0.30*	0.18*	0.26**	-0.02	0.20*
Average air temperature (°C)				0.27*	0.16*	0.28**	-0.09	0.05
SO_2_ (tons/day)					0.65**	0.69**	0.07	0.10
Electric conductivity (μS.cm^-1^)						0.69**	0.29**	0.05
Water temperature (°C)							0.24*	0.11
pH								0.10
Dissolved O_2_ (mg.L-1)								

**p <0.01.

* p< 0.05; the correlations greater than 0.5 and highly significant are bolded.

The result of the cross correlation and univariate model between cholera cases and environmental variables are presented in Table 3. The table shows a significant positive linear association between the number of cholera cases with sulphur dioxide concentration of the volcanic plume, water temperature and conductivity. A value higher than the average of sulphur dioxide concentration of the volcanic plume, temperature and water conductivity was likely to lead to a higher than average number of cholera cases during the same week. Also, the positive effects of sulphur dioxide concentration of the volcanic plume on cholera cases were observed one week after the increase in volcanic activity. Nevertheless, despite a weak correlation (r = 0.37; p = 0.03), the effects of rainfall on cholera cases were better observed four weeks after their increase. A direct effect of water pH on cholera cases was also observed despite the low correlation (r = 0,27, p<0.001; Table 3).

**Table 3:** Cross-correlation coefficients and univariate model of logarithmic transformation of observed number of cholera cases and environmental variables over the 2007–2012 period (n = 147 sampled dates).

Variables			Univariate model		
	Cross correlation lags (weeks)	Correlation coefficients (r)	Odd ratios^1^	95% CI	P-value
Rainfall (mm/week)	0	0.33*	1.01	0.99–1.025	0.36
	1	0.35*	1.01	0.99–1.023	0.3
	2	0.37*	1.02	1.001–1.03	0.03
Average air temperature (°C)	0	0.27*	1.01	0.97–1.17	0.16
	1	0.21*	0.98	0.90–1.08	0.79
	2	0.26*	0.98	0.91–1.07	0.78
SO2 (tons/day)	0	0.76**	1.0004	1.0003–1.0005	< 0.001
	1	0.48**	1.0001	1.00002–1.0002	0.012
	2	0.38**	1.0001	0.98–1.0002	0.07
Water conductivity (μS.cm^-1^)	0	0.75**	1.006	1.005–1.01	< 0.001
	1	0.34**	1	0.99–1.00	0.74
	2	0.30*	1.001	0.99–1.001	0.07
Water temperature (°C)	0	0.80**	1.43	1.35–1.52	< 0.001
	1	0.41**	1.03	9.74–1.10	0.27
	2	0.32*	1.04	0.98–1.039	0.17
pH	0	0.27*	1.36	1.15–1.62	< 0.001
	1	0.03	0.89	0.97–1.065	0.21
	2	0.01	0.98	0.83–1.17	0.87
Dissolved oxygen ([O_2_] mg.l^-1^)	0	0.16	1.08	0.96–1.21	0.165
	1	0.09	0.97	0.86–1.09	0.699
	2	0.04	1.03	0.93–1.14	0.53

^1^ Obtained by exponentiating the estimates obtained from the model

**p <0.01.

* p< 0.05*

Table 4 grouping linear interdependencies among multiple time series using multivariate Vector Autoregression (VAR) models confirmed that volcanic activity influenced the time series of lake parameters (temperature, pH, conductivity) as well as the incidence of cholera cases in the population.

**Table 4:** Linear interdependencies among multiple time series using multivariate Vector Autoregression (VAR) models. Six variables were selected to build this model: cholera cases. rainfall. water temperature. water electric conductivity. pH. volcanic sulphur dioxide emission. For the 2007–2012 period. Katana Halth Zone. DR Congo (n = 147 sampled dates). The significant correlations are bolded.

	variables to explain							
	number of cholera cases				Rainfall (mm/week)			
explanatory variables	Estimate	Std. Error	t-value	Pr (>|t|	Estimate	Std. Error	t-value	Pr (>|t|
number of cholera cases per week					3.082	2.166	1.423	0.157
rainfall (mm/week)	0.003	0.005	0.634	0.527				
pH	0.056	0.083	-0.683	0.496	-1.166	1.099	-1.062	0.290
Water temperature (°C)	0.007	0.066	-0.107	0.914	1.222	0.819	-1.492	0.138
SO_2_ (tons/day)	<0.001	<0.0001	2.133	**0.034**	0.001	<0.001	1.075	0.284
Conductivity (μS.cm-1)	<0.001	0.001	0.639	0.524	0.002	0.016	0.106	0.916
Constant	0.021	1.743	0.012	0.990	37.916	23.191	1.635	0.104
	pH				Water temperature (°C)			
explanatory variables	Estimate	Std. Error	t-value	Pr (>|t|	Estimate	Std. Error	t-value	Pr (>|t|
number of cholera cases per week	-0.309	0.174	-1.777	0.077	-0.075	0.33	-0.227	0.820
rainfall (mm/week)	0.004	0.005	0.860	0.391	0.001	0.01	0.114	0.909
pH					0.024	0.167	-0.146	0.884
Water temperature (°C)	-0.066	0.066	-0.997	0.320				
SO_2_ (tons/day)	<0.001	<0.001	3.342	**0.001**	<0.001	<0.001	2.544	**0.012**
Conductivity (μS.cm-1)	<0.001	0.001	-0.643	0.521	0.003	0.002	1.138	0.257
Constant	8.13	1.866	4.356	0.001	17.527	3.532	4.962	0.0001
	SO2 (tons/day)				Conductivity (μS.cm-1)			
explanatory variables	Estimate	Std. Error	t-value	Pr (>|t|	Estimate	Std. Error	t-value	Pr (>|t|
number of cholera cases per week	429.136	261.85	1.639	0.103	-8.797	16.435	-0.535	0.593
rainfall (mm/week)	0.837	7.881	0.106	0.915	0.598	0.494	1.209	0.229
pH	-125.33	132.816	-0.944	0.347	-10.182	8.336	-1.221	0.224
Water temperature (°C)	-34.056	99.013	-0.344	0.731	-1.937	6.214	-0.312	0.756
SO_2_ (tons/day)					0.0190	0.007	2.574	**0.011**
Conductivity (μS.cm-1)	3.84	1.891	2.030	0.08				
Constant	-651.873	2803.6151	-0.233	0.816	873.845	175.97	4.966	0.001

The instantaneous and Granger-causality tests are simple ways to ascertain whether a variable is affected by changes in other variables. These tests indicate if changes in one variable help forecast a one-step ahead figure in another variable. The test statistics are summarized in Tables 5 and 6. Each column contains the values of F-statistics testing the marginal effect of inclusion of lagged values of the environmental variables in the row on cholera. Both tests indicated that the number of cholera cases was influenced by volcanic activity. In addition, the instantaneous causality tests showed that cholera was influenced by rainfall, Ph, temperature and water conductivity.

**Table 5:** Granger causality tests for the number of cholera cases and environmental variables (rainfall. water temperature. water electric conductivity and pH. volcanic sulphur dioxide emission) for 2007–2012 period. Katana Health Zone. DR Congo (n = 147 sampled dates).

Cause variable	F-value	p-value
SO_2_ (tons/day)	3.219	0.007
Rainfall (mm/week)	0.49	0.783
PH	0.868	0.50
Water temperature (°C)	0.875	0.49
Electric conductivity (μS.cm^-1^)	1.212	0.30

Null hypothesis: environmental variable not Granger cause cholera

Reject the null hypothesis if p-value is < 0.05.

**Table 6:** Instantaneous causality tests for number of cholera cases and environmental variables (rainfall. water temperature. water electric conductivity. water Ph. volcanic sulphur dioxide emission) for 2007–2012 period. Katana Health Zone. DR Congo (n = 147 sampled dates).

Cause variable	Chi-squared-value	p-value
SO_2_ (tons/day)	49.289	0.0001
Rainfall (mm/week)	11.712	0.038
pH	25.68	0.0001
Water temperature (°C)	53.22	0.0001
Electric conductivity (μS.cm^-1^)	51.729	0.0001

Null hypothesis: No instantaneous causality between environmental variable and cholera

Reject the null hypothesis if p-value is <0.05.

**Fig 3:**
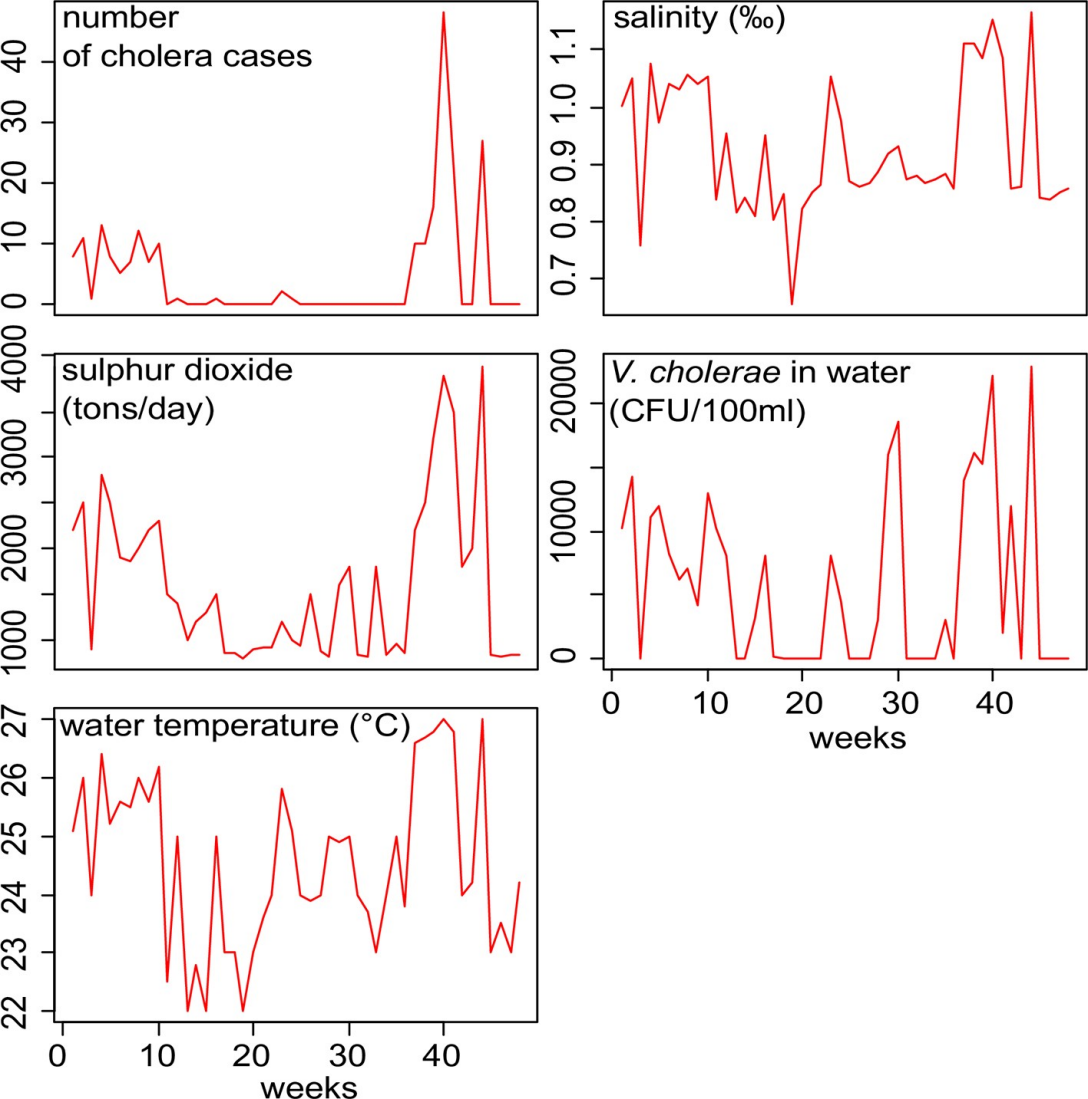
Time series plot of cholera cases, water vibrio occurrence and environmental variables for the period of 2016 to 2017, Katana health zone, DR Congo (n = 48).

Impulse response analysis was utilized to analyse the dynamic interactions between cholera and the volcanic activity of the VAR process. The orthogonal impulse response of cholera to the volcanic activity is presented in Fig 3. The highest positive effect of volcanic activity on cholera is in the fourth week. The positive effect of volcanic activity on cholera incidence is observed from the fourth week and persists until the 15th week, while impulsive responses of volcanic activity on the physico-chemical of the lake are perceptible from the first week.

**Fig 4:**
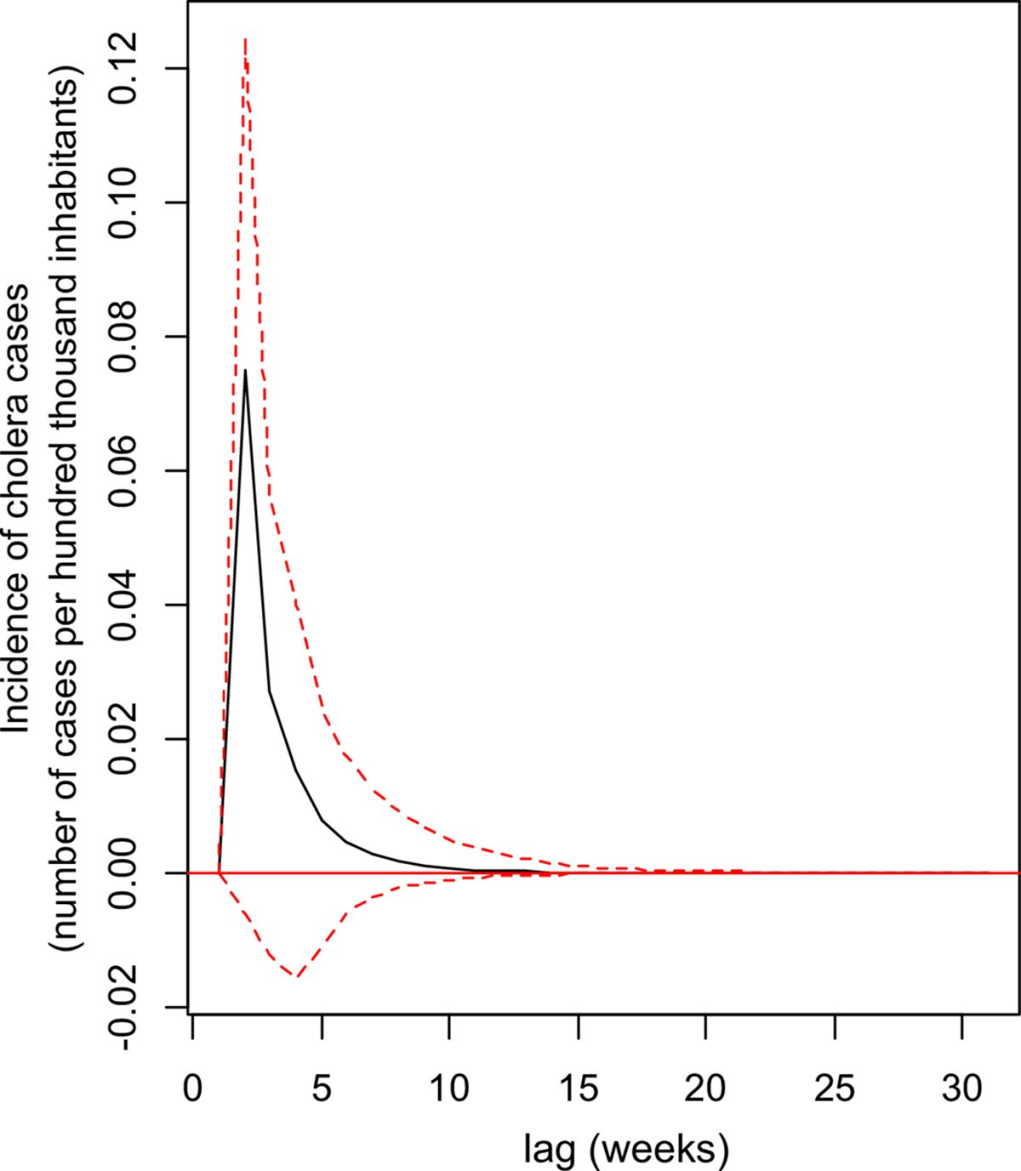
Impulse Response Function (IRF) showing the impact of an increase in volcanic activity (through the flow of volcanic sulphur dioxide) on the incidence of cholera cases in the population of the Katana Health Zone, DR Congo, over a 15-week period. The largest positive effect is observed about three weeks after the shock due to volcanic activity and the return to equilibrium is only observed around the 15th week. Black line: Impulse Response Function, dotted lines: 95% confidence level curves (obtained through 100 runs of the bootstrap method).

The main time series plots (cholera cases, fish contamination, and environmental variables collected from 2016 to 2017) are presented in Fig 4. During the 2016–2017 period, the abundance of *V*. *cholerae* in the lake was positively correlated with lake salinity and temperature and the number of cholera cases in the population. Fish contamination was positively correlated with *V*. *cholerae* abundance in lake water (r = 0,94, p = 0,001) (Table 7). Cross-correlation analyses also showed strong direct correlations between sulphur dioxide concentration of the volcanic plume, temperature and salinity of lake water and the concentration of *V*. *cholerae* in the lake. Also, statistically significant correlations were observed after 2-week lag (Table 8).

**Table 7:** Pearson correlation coefficients between logarithmic transformation of observed cholera cases in the Katana health zone. physico-chemical characteristics of lake Kivu. and the concentration of V. cholerae in lake water and fish over the 2016–2017 period (n = 48 sampled weeks).

Variables	number of cholera cases per week	water temperature (°C)	Salinity (‰)	*V*. *cholerae* in fish (CFU/100 g)	*V*. *cholerae* in water (CFU/100 ml)	SO_2_ (tons/day)
number of cholera cases per week		0.89**	0.90**	0.70**	0.69**	0.89**
Water temperature (°C)			0.93**	0.71**	0.71**	0.77**
Salinity (‰)				0.75**	0.75**	0.82**
*V*. *cholerae* in fish (CFU/100 g)					0.94**	0.77**
*V*. *cholerae* in water (CFU/100 ml)						0.77**
SO_2_ (tons/day)						

**p <0.01.

* p< 0.05; the correlations greater than 0.5 and highly significant are bolded.

**Table 8:** Cross correlations between the concentration of V. cholerae in the Kivu lake. the number of cholera cases in the Katana health zone. and environmental variables over the 2016–2017 period (n = 48 sampled weeks).

Variables	Cross correlation lags (weeks)	Correlation coefficients (r)
SO2 (tons/day)	0	0.77**
	1	0.35**
Salinity (‰)	0	0.74**
	1	0.26**
Water temperature (°C)	0	0.71**
	1	0.27**
Number of Cholera cases per week	0	0.65**
	1	0.32**

**p <0.01.

* p< 0.05

Contamination was significantly higher in intestines than in gills (5% higher in average, linear mixed effect model, permutation test, p < 0.001).

## Discussion

Over the 2007–2012 period, we demonstrated a strong and positive correlation between (1) cholera dynamics, (2) volcanic activity, assessed by sulphur dioxide content of volcano plume, (3) water temperature and conductivity of Lake Kivu and, to a lesser extent, (4) rainfall. More precisely, an above-average rise in volcanic sulphur dioxide modifies the physico-chemical characteristics of the lake in the same week, and the action can persist for several weeks.

There is a poor correlation between air temperature and water temperature, whereas the correlation between volcanic activity and lake temperature is strong, outlining that the lake temperatures are partly controlled, at least during periods of high volcanic activity, by volcanic activity. *V*. *cholerae* usually colonizes warm brackish waters [38, 49], the temperature of which being usually dependent on the climate. In the present situation, volcanic activity may increase the temperature of hydrothermal springs and tributaries in such a way that it leads to favourable thermal conditions for *V*. *cholerae*. Previous studies conducted in hydrothermal areas have demonstrated that lake water temperature increase may lead to the occurrence of cholera cases (e.g. two days after thermal peaks, [50]). Temperature increases are involved in the risk of cholera outbreaks because the abundance of *V*. *cholerae* in water increases as the temperature increases [51, 52]. Under higher temperatures, the growth and multiplication of *V*. *cholerae* might be promoted [53], increasing the risk that the concentration of *V*. *cholerae* in water would be sufficient to be pathogenic if ingested [54, 55].

We demonstrated also an instantaneous causality between the electric conductivity of Lake Kivu water and cholera cases in the population. A study conducted in Lake Victoria and several lakes of the Rift valley demonstrated also a positive relationship between electric conductivity and the number of *V*. *cholerae* colonies in lake waters, but it did not link this relationship to any other environmental variation, nor to epidemiological data [37]. Our results demonstrated for the first time that the strong correlation between water electric conductivity and the occurrence of *Vibrio cholerae* in the lake is linked to environmental factors (namely volcano activity, rainfall, and their possible combined impact on lake water quality), and likely impact the incidence of cholera in the Katana population along the lake shore.

We also demonstrated for the first time that the temporal variation of cholera cases is strongly and at a short time scale, controlled by the temporal variability of volcano activity, together with, in a lesser extent, and in a more delayed way, by rainfall. Indeed, rainfall positively influenced the number of cholera cases 4 weeks after its increase. Heavy rains can provide, through the percolation of volcanic soils in the region, significant quantities of minerals in the lakes, thus promoting bacterial occurrence and the occurrence of cholera outbreaks in the region. The temporal delay between rain peaks and cholera outbreaks may relate to the time required for rainfall to infiltrate in the soil surface, and exfiltrate in the lake tributaries or the lake itself. Rainfall can also affect the growth of the pathogen and its survival. Indeed, high rainfall increases the levels of insoluble iron, which improves the survival of *V*. *cholerae* in aquatic environments. Moderate levels of iron also increase expression of the cholera toxin. It has also been suggested that high rainfall might wash away the vibriophages that prey on *V*. *cholerae* in water, leading to the epidemics of cholera [56, 57].

The significant but low pH positive effect on cholera cases may be due to the known niche of the bacillus, that develop preferably in alkaline waters.

These correlations can reinforce the environmental hypothesis on the endemicity of this region to cholera. Volcanic activity, by modifying the physico-chemical composition of water, may create a niche favourable to the survival of *V*. *cholerae*. These physico-chemical conditions may also have a positive effect on plankton blooms, essential for the growth of *V cholerae* ([58]).

Over the 2016–2017 period, the salinity of the lake water was positively correlated with the occurrence of *V*. *cholerae* in lake water. A positive correlation between the salinity of lake water and the number of cholera cases in the population of the Katana Health zone was also demonstrated. Salinity has been previously demonstrated to be strongly involved in the ecology of *V*. *cholerae* and therefore to contribute indirectly to cholera epidemics [38]. Studies conducted in coastal and estuarine regions in different parts of the world have outlined that temperature and salinity rule partly the occurrence of *V*. *cholerae* in aquatic environments [38, 49].

According to data from the Demographic and Health Survey published in 2014 [59], nearly three out of ten households in the Katana population have access to an improved water source with a regular flow during the year, which explains why the population frequently uses stream and lake water as a drinking water source. Thus, this permanent contact between man and the at-risk lake environment may explain the endemicity of cholera in this area of the rift valley bordering Lake Kivu. The population is indeed chronically exposed to potentially contaminated water. It can therefore be realistically hypothesized that it is variations in vulcanological functioning and associated hydrothermalism that govern the degree of contamination of surface waters, and the epidemiology of *V*. *cholerae*.

We also demonstrate a significant and positive link between cholera dynamics in the population and fish and water contamination. The fish caught in the lake have a bacillus load that is highly correlated with the load in lake water. Depending on the studies, *Vibrio* may be found [60] or not [61]), but the studies usually not try to correlate the occurrence of *Vibrio* simultaneously in the water and in the animals (but see [62] for example). The local residents frequently eat these fish raw or salted, without emptying them nor cooking them. These consumption practices can consequently increase the risk of consumer contamination [63]. It is also possible that the bacillus survives at least for a few days, in salted food [64–66]. Contaminated fish may remain sufficiently wet for maintaining alive some *V*. *cholerae* and may therefore be a source of contamination for consumers for several days after collection. Transport and trade of salted or fresh fishes to other provinces may then spread the bacillus to preserved populations.

The findings of this study consistently support, for the first time, the idea that volcano-tectonic activity, by largely controlling the hydrothermal functioning of Lake Kivu, determines the physico-chemistry of the lake and consequently likely induces cholera outbreaks. The correlations are so strong that the volcanic signal can be used as an environmental predictor of cholera blooms in the lake and thus as an alert signal for populations.

The Rift paradox concerning cholera occurrence along saltwater lakes of the Rift [12, 24, 31] can be explained by the salinization and warming of water as volcanic activity increases. The high variability of *Vibrio cholerae* contamination in a series of large lakes in the African rift recently observed may relate to the hydro-geological and volcano-tectonic context of each lake [67]. These factors would control the physico-chemical characteristics of each lake, and therefore their susceptibility to constitute habitats for *Vibrio cholera* [67]. Consequently, the bacillus niche does not appear to be significantly different from that described in previous studies in coastal areas [2, 3, 5, 6, 68], such as the lagoon areas of India [4].

However, the question of when and how cholera appeared in the rift valley remains open. The first cases of cholera in the Great Lakes region of Africa were observed in 1971, mainly in Kenya and then in Uganda [69, 70]. In a report from September 1971, the "Belgian cooperation mission" was already concerned about the risk of cholera in the eastern Congolese region. For this reason, the mission decided to send 20 kg of cholera vaccines [69, 71]. The part of the Great Lakes located in the present Democratic Republic of Congo (DRC), then called Zaïre, was affected only in September 1977, whereas the first epidemic had already occurred (in 1974) in the littoral part of the country (province of Kongo Central, then called Bas Zaïre) more than 1700 km away, in the lakeside city of Kalemie, located on on the west right bank of Lake Tanganyika (water flows from the north to the south in this part of the rift chain of lakes). From this point on, the disease spread rapidly throughout the eastern part of the DRC. The 1978 epidemic in the eastern DRC (In Kalemie, on the right bank of Lake Tanganyika), originated from Tanzania and concentrated near the Great Lakes. On this occasion, contaminated water was proven to play a role in the transmission of the disease [72].

The Nyiragongo volcano has erupted only twice (10^th^ of January 1977 and 17^th^ of January 2002) since it has been monitored [28]. Both eruptions were preceded by the appearance of mineral sources close to the city of Sake (NW Kabuno Bay), indicating the circulation of water in the fracture system just before the eruptions [28]. Hydro volcanism also occurred in the past in the southern part of the main NE-SW fracture system [27, 30, 73, 74], revealing the strong coupling between water circulation of the recharging aquifer system and fractures in the area close to the northern shoreline of Kivu Lake [30, 33]. Sub-groundwater discharges into the Northern part of the lake likely occurred along these faults and fractures, which are also involved in the structuration of the Kivu rift segment, and which may connect aquifers over different depths [29]. The acidic gases (HF, HCl, CO_2_, and SO_4_^2-^) of the eruptive plume of Nyiragongo volcano are highly soluble in water and generate a low pH, which favours the dissolution of volcanic ash and may also affect the composition of rainwater and ultimately of rivers reaching the lake [23, 75]. This plume indirectly contributes to an increase in the temperature and salinity of the surface waters of Lake Kivu, which is favourable to the survival of *V*. *cholerae*. *Ad minima*, the reactivation of volcanic activity has led to an increase in hydrothermal inputs to the lake, a factor affecting thermal increase and salinization.

### Conclusion

Cholera is a serious public health problem in the health zones located along the Rift in general and more particularly in the Katana health zone. The high exposure of this population due to limited access to safe water raises an urgent need for new and effective cholera prevention strategies. This study offers a unique insight by demonstrating in a health zone of Lake Kivu, the direct influence of volcanic activity on water physico-chemical characteristics and *V*. *cholerae* occurrence and abundance in the lake water and fish, and thus on the cholera dynamics in the population. These elements of understanding are therefore for public health authorities to readjust operational plans for cholera control in this region. Interventions that target vital steps in transmission might be effective for the prevention of outbreaks. The Nyiragongo volcanic activity and the Kivu lake physico-chemical characteristics must be considered as risk factors and the monitoring of these environmental factors must be integrated into the prevention devices for preventing contamination risk and limiting cholera epidemics. In other words, while maintaining efforts to achieve an acceptable level of access to drinking water for the population, the monitoring of volcanic activity as well as lake physico-chemical characteristics to improve cholera surveillance in the region as part of the multisectoral cholera elimination plan. There are some limitations however to this study. The first one is that we only focused on one health zone, at the immediate proximity of the volcano. It is still necessary to determine the way of human contamination of the other health zones bordering the lake. Is it due to human contamination, or through the consummation of contaminated food and water? In this last hypothesis, is there a delay in the epidemic response, related to the circulation of water in the lake, which is drained by the Ruzizi River? For answering this question, new investigations including other health zones along the lake may be necessary, including epidemiological data, together with water quality and fish contamination surveys.

The failure to achieve PCR in this study is a limitation in determining vibrio toxicity genes. Indeed, only serogroups O1 and O139 are the cause of epidemics, while non-O1 and O139 cause mild diarrhoea. However, previous studies have demonstrated that toxigenic serogroups O1 are abundant in this cholera epidemic sanctuary (Katana Health Zone: [76]). Further studies on the conserved strains will be necessary to identify precisely which serogroups are present in lake surface water and fish, and to determine whether they may respond differently to environmental variations.

In the same way, many questions remain opened on the geological mechanisms leading to the control of physico-chemical characteristics of lake water, such as the direct effect of volcanic gas and tephra. In the same wat, the question of the parameters directly involved in *V*. *cholerae* multiplication (and particularly the part of physico-chemical and trophic controls) in the lake needs to be identified. Finally, the study shows the disappearance of *V*. *cholerae* from surface waters in the Katana sector during periods of low volcanic activity, suggesting refuges for the vibrio in the lake. One hypothesis would be that these refuges are located at depth, close to hydrothermal springs, which implies a vertical migration of the bacillus when the temperature and salinity of the surface waters increase.

## Supporting information

S1 Table Weekly data collected over the 2007–2012 period: the number of cholera cases in the Katana health zone, meteorological data, physico-chemical characteristics of the Kivu lake water (Ishungu station in the Katana Health zone), and the SO2 flux of the Nyiragongo volcano plume. (XLSX)

S2 Table *Vibrio cholerae* abundance in fishes (intestine and gills) collected twice a month in Lake Kivu (Ishungu station in the Katana Health zone) over the 2016–2017 period. (XLSX)

S3 Table Water characteristics measured twice a month in the Kivu lake (Ishungu station in the Katana Health zone) over the 2016–2017 period: temperature, salinity (PSU), SO2 flux of the Nyiragongo volcano plume and abundance of Vibrio cholerae in surface water. The number of cholera cases in the Katana health zone counted twice a month over the same period is indicated.
(XLSX)
